# Grading Medical Students During their Fourth Year Orthopaedic Surgery Rotations

**DOI:** 10.15694/mep.2017.000133

**Published:** 2017-07-20

**Authors:** Joey P. Johnson, Niketu Patel, Patrick Wang, Mary Mulcahey

**Affiliations:** 1 Brown University; 2 Drexel University; 3 Tulane University

**Keywords:** Away Rotations, Grading, Orthopedics

## Abstract

This article was migrated. The article was marked as recommended.

Introduction

An orthopaedic surgery clerkship is an important aspect of a medical student’s application for residency, however, the process by which grades in these clerkships are determined is poorly understood. In this study, we sought to determine what grading systems are being used by orthopaedic residency programs and some of the factors taken into account when grading rotating medical students.

Methods

A 24-question anonymous survey was distributed to residency coordinators at ACGME accredited civilian and military United States orthopaedic surgery residency programs. The survey included questions about the grading system, number of rotating students and factors considered when determining a student’s grade. Standard descriptive statistics were used to evaluate survey responses.

Results

At 57% (16/28) of programs that responded, more than 40% of current residents rotated as 4 ^th^ year medical students. 50% of orthopaedic surgery programs who responded to the survey indicated that 80%-100% of away and home rotating students over the past 5 years received a grade of honors.

Discussion

The current process of grading orthopaedic surgery away rotations suggests that grades may be inflated, with more students receiving the highest designation than are truly deserving of that honor. Standardized grading of these rotations may benefits programs and students alike.

## Introduction

Orthopaedic surgery is one of the most competitive subspecialties in the residency match. As of 2014, there were almost 1.5 applicants per orthopaedic surgery resident position [ NRMP 2014] ^1^. One challenging aspect of matching into orthopaedic surgery residency is the lack of understanding of selection criteria. This leaves students interested in orthopaedic surgery uncertain about what aspects of their application carry the most weight. Previous studies highlighted three criteria that program directors use when selecting applicants for an interview: 1) whether the candidate performed a rotation at that institution, 2) the candidate’s United States Medical Licensing Examination (USMLE) Part 1 score, and 3) the candidate’s medical school class rank [ Bernstein et al. 2016] ^2^.

In a study of clerkship grading in U.S. Medical schools, Alexander et al., found significant variation in grading of medical students during their clerkships [ Alexander et al. 2012] ^3^. The authors demonstrated that medical schools across the nation use grading systems ranging from a 2-tiered to an 11-tiered system [ Alexander et al. 2012] ^3^. Within these systems, there was a significant discrepancy in grading terminology; for example, some schools define honors as the top grade, while others define it as only second best grade [ Alexander et al. 2012] ^3^. Even within similar systems (e.g. the standard A through F scale) there was high variability, as some schools would incorporate a plus/minus system, while others only use the plus aspect [ Alexander et al. 2012] ^3^. Additionally, they demonstrated that the higher the number of tiers in a grading system, the more skewed the grades of rotators. ^3^ The authors showed that a wider range in grades leads to a more skewed distribution. In grading systems with four tiers or more, a higher proportion of students received the top honor [ Alexander et al. 2012] ^3^. Of all students in this survey, 97% received one of the top three grades, regardless of the type of grading system used [ Alexander et al. 2012] ^3^. Conversely, another study found that third year surgery clerkships gave the fewest number of students the highest grade when compared to other specialties [ Takayama 2006] ^4^. The lack of a standardized grading system, and variation in the way grading systems are implemented, makes it difficult to compare applicants based on rotation grades at various institutions.

Although all orthopaedic surgery residency programs provide a grade for rotating students, there is little understanding of the specific criteria that are taken into account when formulating that grade. A study by Zahn et al. in 2004, evaluated grading practices for obstetrics and gynecology clerkships and found that there was “no universally accepted curriculum development or assessment process” [ Zahn et al. 2004] ^5^. The authors found that programs often use history and physical examinations with written notes, patient presentations, group discussions, and ward rounds as subjective criteria for grading students, while performance on National Board of Medical Examiners exams is often incorporated as an objective means of grading [ Zahn et al. 2004] ^5^. There is also considerable variability among institutions in how much each aspect contributes to the final grade, with some OB/GYN rotations using the NBME exam for as little as 40% of the final grade, while other programs weighing it as much as 70% [ Zahn et al. 2004] ^5^.

During the fourth year of medical school, students interested in competitive subspecialties (e.g. orthopaedic surgery, plastic surgery, neurosurgery) will often participate in rotations at outside institutions. Away rotations benefit the applicant by familiarizing him/her with teaching faculty and residents at a given institution and providing the applicant with the opportunity to impress these individuals. These rotations also allow students to obtain letters of recommendation from orthopaedic staff members, which could bolster the applicant’s chances of matching at that institution. ^6^ Away rotations benefit residency programs by giving faculty and residents a more personal and in-depth look at each applicant beyond what is provided on his or her application. During the rotation, faculty and residents have the opportunity to observe an applicant’s work ethic, bedside manner, interactions with ancillary staff, and personality in order to determine if they are an appropriate fit for the program. Previous studies have demonstrated that completion of a 4 ^th^ year orthopaedic surgery rotation is one of the most important criteria in the selection of residents. In a study by Bernstein et al, in 2004, orthopaedic surgery residency program directors reported that over half of the residents in their program had rotated at their institution as medical students [ Bernstein et al 2016] ^2^. A separate study demonstrated that students who had performed two away rotations were more likely to match than students who did only one away rotation or none at all [ Camp et al. 2016] ^7^.

The purpose of our study is to determine the factors taken into account when grading medical students during their fourth year orthopaedic sub-internship and to assess the value of the grade obtained. We hypothesize that grades given during an orthopaedic surgery rotation are inflated, with more students receiving the highest designation than may be deserving of that honor.

## Methods

Our Institutional Review Board (IRB) determined no formal IRB approval was necessary for this study. A 24-question survey regarding factors taken into account when grading 4 ^th^ year medical students rotating at orthopaedic surgery residency programs was distributed via Google Forms to the residency coordinators of the 146 United States ACGME accredited orthopaedic surgery residency programs and four U.S. Military orthopaedic surgery residency programs, for a total of 150 programs. Osteopathic and non-ACGME accredited orthopaedic surgery residency programs were excluded.

The link to the anonymous survey was sent via email, along with a description of the study. Email addresses for the residency coordinators were collected by the research team using publicly accessible information found on each respective residency program website. A follow-up email was sent two and four weeks after the initial message to encourage more participation. The survey included questions about the grading system, the number of home and away students rotating, grade distributions, and demographics (Appendix A). Standard descriptive statistics were used to analyze survey responses.

## Results

Of the 150 United States ACGME accredited allopathic orthopaedic surgery residency coordinators contacted (146 civilian and 4 military), 11 emails were unsuccessfully delivered. Our statistics were therefore based on the 139 programs that successfully received the survey. In total, 28 of 139 (20% response rate) orthopaedic surgery residency programs responded to the survey.

Of the responding programs, 75% (21/28) have a grading system for their orthopaedic clerkship that includes honors / high pass / pass / fail. Eight out of 28 (29%) programs also report not using the same grading system for home and away rotators. At 13/28 (46%) programs, more than 50% of 4 ^th^ year medical students rotating are from outside institutions. Half of the programs reported awarding 80%-100% of their home students a grade of honors, with another 25% (7/28) of programs giving between 60%-79% of home students honors. This trend was mirrored by grades awarded to visiting students, with 54% (15/28) of programs awarding between 80%-100% of the visiting students a grade of honors, while 21% of the programs giving between 60%-79% of their visiting students honors.

When considering an applicant for orthopaedic surgery residency, 79% (22/28) of programs felt that the grade received during an orthopaedic clerkship was of high importance. One out of 28 (4%) programs thought it was neutrally important, and 14% thought it was of low importance, with one program not providing a response. When asked how important it was for the applicant to have participated in an orthopaedic clerkship at their institution, 46% (13/28) rated it as highly important, while 32% (9/28) rated it as neutrally important, and 21% (6/28) rated it as low importance. The percentage of current residents that performed a fourth-year clerkship at their place of residency is summarized in Table 2.

Programs ranked the following factors as “high importance” for grading a student during their orthopaedic rotation: enthusiasm and interest (100%), professionalism (96%), student relationship with the residents (93%), and knowledge base (89%). Of the programs responding, 57% rated surgical skills as highly important, with 29% determining it to be neutral, and 14% as low importance. The quality of case presentations was considered to be of high importance by 64% of programs, neutral importance by 14% of programs, low importance by 7% of programs, and the remaining 15% of programs did not rank this factor ( Table 1). A student’s interactions with ancillary staff (administrative assistant, OR staff, etc) was rated highly important by 79% (22/28), neutral by 18% (5/28), and of low importance by 4% (1/28) of programs.

Regarding the demographics of the orthopaedic residency programs, 79% (22/28) of programs reported having more than 8 total residents, over all four years of training. For more than half of the programs, less than 15% of their current residents are women ( Table 3).

## Discussion

Away rotations in orthopaedic surgery are an important part of the process of applying for orthopaedic residency, however, there is significant variability in grading practices between programs. Since most medical students receive a grade of honors on their rotation, this raises the question as to whether this is a reliable way to differentiate students. In general, a grade of honors is reserved for the select few who adequately demonstrate competency and skills above and beyond the rest of their peers. This was not reflected in the data from our study. Half of programs gave 80%-100% of their home students a grade of honors, with another quarter of programs giving between 60%-79% honors. The grades received by visiting students were similar, with most programs giving a grade of honors to at least 60% of away students. With nearly three quarters of programs giving more than half of their home and away students a grade of honors, it suggests that a grade of honors may at times be awarded regardless of the student’s performance.

Despite the questionable value of the Honors designation, accredited orthopaedic surgery residency programs continue to place a lot of value on the grade a student receives during the orthopaedic rotation. This is evidenced by 79% of programs regarding the grade received during an orthopaedic clerkship as highly important. This indicates that most orthopaedic programs still view the grade as a good indicator of a student’s orthopaedic competency.

A study by Depasse et al demonstrated that orthopaedic surgery residencies are becoming increasingly competitive [Depasse et al. 2014] ^8^. The authors found that although match rates have not changed over a 7-year period, applicants are becoming more qualified. This study demonstrated a significant increase in USMLE Step 1 scores during this time period for successfully matched applicants [Depasse et al. 2014] ^8^. Additionally, the authors found that applicants who matched into orthopaedic surgery had on average doubled their number of scientific publications from 3 publications in 2007 to 6 publications in 2014 [Depasse et al. 2014] ^8^. This demonstrates that the pool of potential orthopaedic surgery residents is comprised of highly qualified medical students, and it is becoming incredibly difficult for applicants to separate themselves from each other [Depasse et al. 2014] ^8^. The results of our study further support the increasingly competitive process of applying for orthopaedic surgery residency.

Given the competitive pool of applicants for orthopaedic surgery residency positions, fourth year orthopaedic clerkships remain a vital way for applicants to stand out from their peers. Bernstein et al. surveyed orthopaedic residency program directors and found that the most critical factor for an applicant to successfully match at an orthopaedic program was whether the applicant had rotated at that institution [ Bernstein et al. 2016] ^2^. Additionally, a study by Baldwin et al., found a positive correlation between the number of away rotations a student had completed and their likelihood of matching [ Baldwin et al. 2009] ^6^. The authors highlighted the importance of applicants being able to get letters of recommendation and having a chance to demonstrate their work ethic and knowledge base during these rotations [ Baldwin et al. 2009] ^6^. Similarly, Camp et al. showed that completing an away rotation at a given orthopaedic residency program increased an applicant’s chance of matching at that program by 150% [ Camp et al. 2016] ^7^. The importance of a student’s performance on these clerkships was further supported in our study as a student’s enthusiasm/interest and professionalism ranked as two of the most valued categories by programs (100% and 96%, respectively).

In our study, surgical skills were ranked as highly important by 57% of responding programs, demonstrating that inherent surgical skill among medical students is considered to be important, but not crucial. This is likely because surgical skill can be learned with practice and should not necessarily be used to gauge the technical skill a student will be able to obtain as an orthopaedic resident. However, inherent surgical skill in a medical student may speak to their ability to quickly learn more complex skills as they progress through training. Additionally, proficiency in the operating room may indicate that a medical student understands his/her role in that setting, a skill that can be quite valuable for a resident. Perhaps most importantly, surgical skill is something that can be identified while an applicant is on a visiting clerkship, and may be part of the reason why the literature widely supports the importance of these rotations [ Bernstein et al. 2016] ^2^ [ Baldwin et al. 2009] ^6^ [ Camp et al. 2016] ^7^ [ O’Donnell et al. 2017] ^9^.

Several studies have demonstrated the value of away rotations as a way of supplementing the paper application and allowing applicants to determine if a specific program is a “good fit” [ Bernstein et al. 2016] ^2^ [ Baldwin et al. 2009] ^6^ [ Camp et al. 2016] ^7^. Given the emphasis placed on these rotations by both applicants and residency programs, a textbook was recently published detailing the importance of the rotations and providing suggestions on how to stand out during the clerkship [ Eltorai et al. 2016] ^10^. The goal of this textbook was to clarify for applicants important details about orthopaedic rotations including how and where to apply and how to find information regarding different programs [ Eltorai et al. 2016] ^10^. However, the grading algorithms associated with these programs was not addressed given the substantial variability between programs [ Eltorai et al. 2016] ^10^. Our study further supports the value of the away rotation in the process of applying for orthopaedic residency. Of programs responding, 46% rate the away rotation as a highly important component of a medical student’s application for residency. More than 20% of current orthopaedic residents at 86% of programs rotated at that program as a fourth-year medical student, which further underscores the emphasis placed on these rotations. These numbers seem to mirror the reported rate of medical students who perform an away rotation in plastic surgery and then match into that specialty [ Drolet et al. 2016] ^11^. Drolet et al. reported that 44% of PGY1 plastic surgery positions were filled by students who had rotated at that institution (27% visiting students and 17% home students) [ Drolet et al. 2016] ^11^.

There are several limitations to this study including the low response rate (28 of 139, 20%), which may limit the generalizability of the results. Also, since individual evaluations for rotating medical students were not accessible, we cannot comment specifically on the performance of each student, or how our survey responses correlate to the awarding of actual clerkship grades. Additionally, the surveys were distributed to the residency coordinators who may not be aware of the full grading algorithm. In the information letter sent to the coordinators, we requested that the survey be forwarded to the clerkship director if the coordinator himself/herself was not familiar with the grading practices, however, there is no way for us to know if this occurred.

The 4 ^th^ year medical student away rotation in orthopaedic surgery plays an important role in the application process for residency, for both the applicant and the residency programs. Studies demonstrate the increasing qualifications of applicants to orthopaedic residency, however, there needs to be a method to differentiate between these outstanding applicants while they are rotating at given institutions [ Depasse et al. 2016] ^8^. As it currently stands, the grade a student receives on the rotation may not be helpful in differentiating an individual student’s performance from that of his/her peers. The results of this study support the need for change and standardization of grading practices for the orthopaedic surgery clerkship. Objective grading systems, if universally adopted, would allow for orthopaedic surgery clerkship grades to have a larger impact on a student’s application, especially when being evaluated by faculty at programs where the student did not rotate.

**Table 1.** Factors influencing orthopaedic surgery rotation grades.

	High Importance	Neutral Importance	Low Importance
**Enthusiasm and interest**	100% (28/28)	0% (0/28)	0% (0/28)
**Professionalism**	96% (27/28)	4% (1/28)	0% (0/28)
**Relationship with residents**	93% (26/28)	7% (2/28)	0% (0/28)
**Eagerness to help residents**	93% (26/28)	7% (2/28)	0% (0/28)
**Knowledge base**	89% (25/28)	11% (3/28)	0% (0/28)
**Interactions with ancillary staff**	79% (22/28)	18% (5/28)	4% (1/28)
Quality of case presentations *	64% (18/28)	14% (4/28)	7% (2/28)
**Surgical skills**	57% (16/28)	29% (8/28)	14% (4/28)

* 15% (4/28) of programs did not rank case presentations at all, likely because it was not applicable to their institution

**Table 2.** Percentage of current residents that performed a fourth-year clerkship at their residency program.

Percentage of Residents That Rotated	Percentage of Programs
0-19%	(4/28) 14%
20-39%	(8/28) 29%
40-59%	(10/28) 36%
60-79%	(4/28) 14%
80-100%	(2/28) 7%

**Table 3.** Percentage of current residents that are female.

Percentage of Female Residents	Percentage of Programs
<5%	(6/28) 21%
5-9%	(5/28) 18%
10-14%	(6/28) 21%
15-19%	(7/28) 25%
20%+	(4/28) 14%

## Take Home Messages

• Orthopaedic surgery clerkships are a vitally important portion of a medical student’s application
• The grading systems for orthopaedic surgery clerkships, are highly variable and likely artificially inflated
• Standardization of orthopaedic surgery clerkship grading would allow for these grades to be objectively scrutinized as part of an applicant’s application

## Notes On Contributors

Joey P. Johnson, MD is a Trauma Fellow in the Department of Orthopaedic Surgery at Brown University.

Niketu Patel, B.S. is a medical student at Drexel University College of Medicine.

Patrick Wang, MD is a resident in the Department of Orthopaedic Surgery at Drexel University.

Mary K. Mulcahey, MD is an Associate Professor and the Director of the Women’s Sports Medicine Program in the Department of Orthopaedic Surgery at Tulane University.

## Appendices

Assessing the Grading Practices for 4th Year Orthopaedic Surgery Rotations

1. What grading system do you use at your institution for grading orthopedic surgery rotations?

• Honors / High Pass / Pass / Fail
• A / B / C / D / F
• A+ / A / A- /B+ / B / B- / etc.
• Pass / Fail
• Other (Please describe):

2. Is the grading system the same for home or away rotators?

• Yes
• No

3. On average, how many total fourth year students rotated in the Orthopedic Surgery Department at your institution each year for the past 5 years?

• <10
• 10-15
• 15-20
• 20-25
• >25

4. What percentage of students were away rotators each year during that 5 year period?

• <10%
• 10-20%
• 20-30%
• 30-40%
• 40-50%
• > 50%

5. On average, what percentage of your overall residents for the past 5 years have been women?

• 0-5%
• 5-10%
• 10-15%
• 15-20%
• >20%

6. Over the past 5 years, what is the average percentage of home rotators who have received a grade of Honors (or equivalent)?

• 0-20%
• 20-40%
• 40-60%
• 60-80%
• 80-100%

7. Over the past 5 years, what is the average percentage of away rotators who have received a grade of Honors (or equivalent)?

• 0-20%
• 20-40%
• 40-60%
• 60-80%
• 80-100%

8. Over the past 5 years, what is the average percentage of home rotators who have received a grade of pass (this should only include those individuals who earned a grade of ‘pass’, and not students who earned a grade lower or higher than ‘pass’)?

• 0-20%
• 20-40%
• 40-60%
• 60-80%
• 80-100%

9. Over the past 5 years, what is the average percentage of away rotators who have received a grade of pass (this should only include those individuals who earned a grade of ‘pass’, and not students who earned a grade lower or higher than ‘pass’)?

• 0-20%
• 20-40%
• 40-60%
• 60-80%
• 80-100%

10. Which of the following factors do you consider to be most important in determining the grade each student receives? Use the following scale to rate each criteria:

No importance 1 2 3 4 5 Most Important

**CriteriaRating**

Professionalism

Interest/Enthusiasm

Getting along with current residents

**CriteriaRating**

Eagerness to help the residents

Knowledge base

NBME Shelf Examination

Quality of surgical skills

Quality of student case presentation (if applicable)

Interactions with ancillary staff (administrative assistant, OR staff, etc)

11. Are the factors used to determine the grade each student receives different for away rotators?

• Yes
• No

12. Have the criteria used to grade your rotators changed in the past 5 years?

• Yes
• No

13. When considering an applicant for an orthopedic surgery residency, how important is the grade received on the 4th year orthopedic rotation?

Not important | 1 2 3 4 5 | Very important

14. When considering an applicant for an orthopedic surgery residency, how important is it for the applicant to have rotated at your institution?

Not important | 1 2 3 4 5 | Very important

15. How many total orthopedic surgery residents are in your program?

• <4
• 4-6
• 6-8
• >8

16. What percentage of your current orthopedic surgery residents rotated at your institution in their fourth year of medical school?

• 0-20%
• 20-40%
• 40-60%
• 60-80%
• 80-100%
