# Supplementary material for: Grading Medical Students During their Fourth Year Orthopaedic Surgery Rotations
Source: MedEdPublish (2016). 2017 Jul 20;6:133. [Version 1] doi: 10.15694/mep.2017.000133 (PMC10885282; doi:10.15694/mep.2017.000133)
Supplement: Peer review history

# Reviewer report: Reviewer response for version 1

**DOI:** 10.21956/mep.19320.r28197

**Published:** 2017-07-26

**Authors:** Linda Nield

**Affiliations:** 1 West Virginia University School of Medicine

This review has been migrated. The reviewer awarded 4 stars out of 5 Well written. With the increasing debt of medical students in the US, it is disconcerting that such emphasis is placed upon students needing to do away rotations (which can be costly to attend).Two edits needed for the work:1. The word "benefits" should be changed to "benefit" in the last sentence of the abstract.2. In paragraph 3 of the Introduction, the authors should indicate that "NBME" is the abbreviation for "National Board of Medical Examiners," as the abbreviation is used in the last sentence of that section.One suggestion:1. If not included, the authors could have added a question to the survey to determine who actually completed the survey, such as:Who is completing this survey?Program Coordinator, Program Director, Associate Program Director, Chief Resident, Other(ideally to have had it completed by the personnel most knowledgeable about how grades are determined)

Reviewer Expertise:

NA

No decision status is available

# Reviewer report: Reviewer response for version 1

**DOI:** 10.21956/mep.19320.r28196

**Published:** 2017-07-22

**Authors:** John Cookson

**Affiliations:** 1 University of Worcester

This review has been migrated. The reviewer awarded 4 stars out of 5 This study provides further evidence of the problems selecting students and qualified doctors into career pathways. The variety of methods used and the grading systems within them suggest that programme directors in orthopaedics have little consensus on what is important. There is no reason to suppose that other specialties are any different. Standardisation might help but first we need to know what factors predict success (or failure), otherwise we are working in the dark. Further, standardisation has its own problems in reducing discussion, debate and experimentation. Long term follow-up on the career progress of candidates on a national basis might help to define what is important.

Reviewer Expertise:

NA

No decision status is available

# Reviewer report: Reviewer response for version 1

**DOI:** 10.21956/mep.19320.r28198

**Published:** 2017-07-20

**Authors:** Trevor Gibbs

**Affiliations:** 1 AMEE

This review has been migrated. The reviewer awarded 4 stars out of 5 An interesting paper that is US -centric regarding the importance of orthopaedic surgery, but never the less adds to our debate as to the value of grading a competency-based specialty , perhaps using normative figures to provide grades and the value of direct observation, standardisation of competencies and using other value systems ( sometimes described as the softer values) to assign these grades. . We need to constantly evaluate whether an honours level at a certain subject actually makes them a better holistic practitioner in that specialty.I am not sure that such away type of rotations can be classed as artificially inflated, its possible that if there are no standards created then the assessors are measuring something different.I think that this paper can attract a lot of debate and would consider it to be an important one for all assessors in higher medical education, despite the reservations explored

Reviewer Expertise:

NA

No decision status is available
